# Advances in urethral stricture management

**DOI:** 10.12688/f1000research.9741.1

**Published:** 2016-12-23

**Authors:** Maxx A. Gallegos, Richard A. Santucci

**Affiliations:** 1 The Center for Urologic Reconstruction, Detroit, MI, USA

**Keywords:** Urethral stricture, Urethral stenosis, Urethroplasty, Spongiofibrosis, Internal Urethrotomy

## Abstract

Urethral stricture/stenosis is a narrowing of the urethral lumen. These conditions greatly impact the health and quality of life of patients. Management of urethral strictures/stenosis is complex and requires careful evaluation. The treatment options for urethral stricture vary in their success rates. Urethral dilation and internal urethrotomy are the most commonly performed procedures but carry the lowest chance for long-term success (0–9%). Urethroplasty has a much higher chance of success (85–90%) and is considered the gold-standard treatment. The most common urethroplasty techniques are excision and primary anastomosis and graft onlay urethroplasty. Anastomotic urethroplasty and graft urethroplasty have similar long-term success rates, although long-term data have yet to confirm equal efficacy. Anastomotic urethroplasty may have higher rates of sexual dysfunction. Posterior urethral stenosis is typically caused by previous urologic surgery. It is treated endoscopically with radial incisions. The use of mitomycin C may decrease recurrence. An exciting area of research is tissue engineering and scar modulation to augment stricture treatment. These include the use of acellular matrices or tissue-engineered buccal mucosa to produce grafting material for urethroplasty. Other experimental strategies aim to prevent scar formation altogether.

## Introduction

Urethral stricture is defined as a narrowing of the urethra. The urethral mucosa is enveloped by corpus spongiosum. This blood-rich erectile tissue surrounds the urethra from the meatus to the bulbar urethra. As the spongiosum provides the vascular supply to the urethra, the degree of fibrosis in corpus spongiosum relates directly to the extent and severity of the stricture. This scar formation is progressive and is called spongiofibrosis ^1^. A urethral stricture is formed when the spongiosal tissue is replaced by dense non-elastic collagen fibers interspersed with fibroblasts ^2^. There are a variety of insults which incite fibroblastic changes to the urethra. These include inflammatory causes such as infections or lichen sclerosus and traumatic causes such as iatrogenic injury or pelvic fracture.

A nomenclature distinction is worth noting: when scar tissue forms in the posterior urethra at the bladder neck, prostatic urethra, and membranous urethra, there is no corpus spongiosum. This is defined as urethral *stenosis* or as bladder neck contracture when it involves that structure.

Urethral strictures are responsible for 5,000 hospital and 1.5 million office visits per year in the US ^3^. Urethral stricture incidence is between 200 and 1,200 cases per 100,000 men/year, although the incidence increases with age ^3^. Estimated annual healthcare costs for male urethral stricture management in the US were $191 million in 2000 ^3^ and are surely higher now.

**Figure 1.**
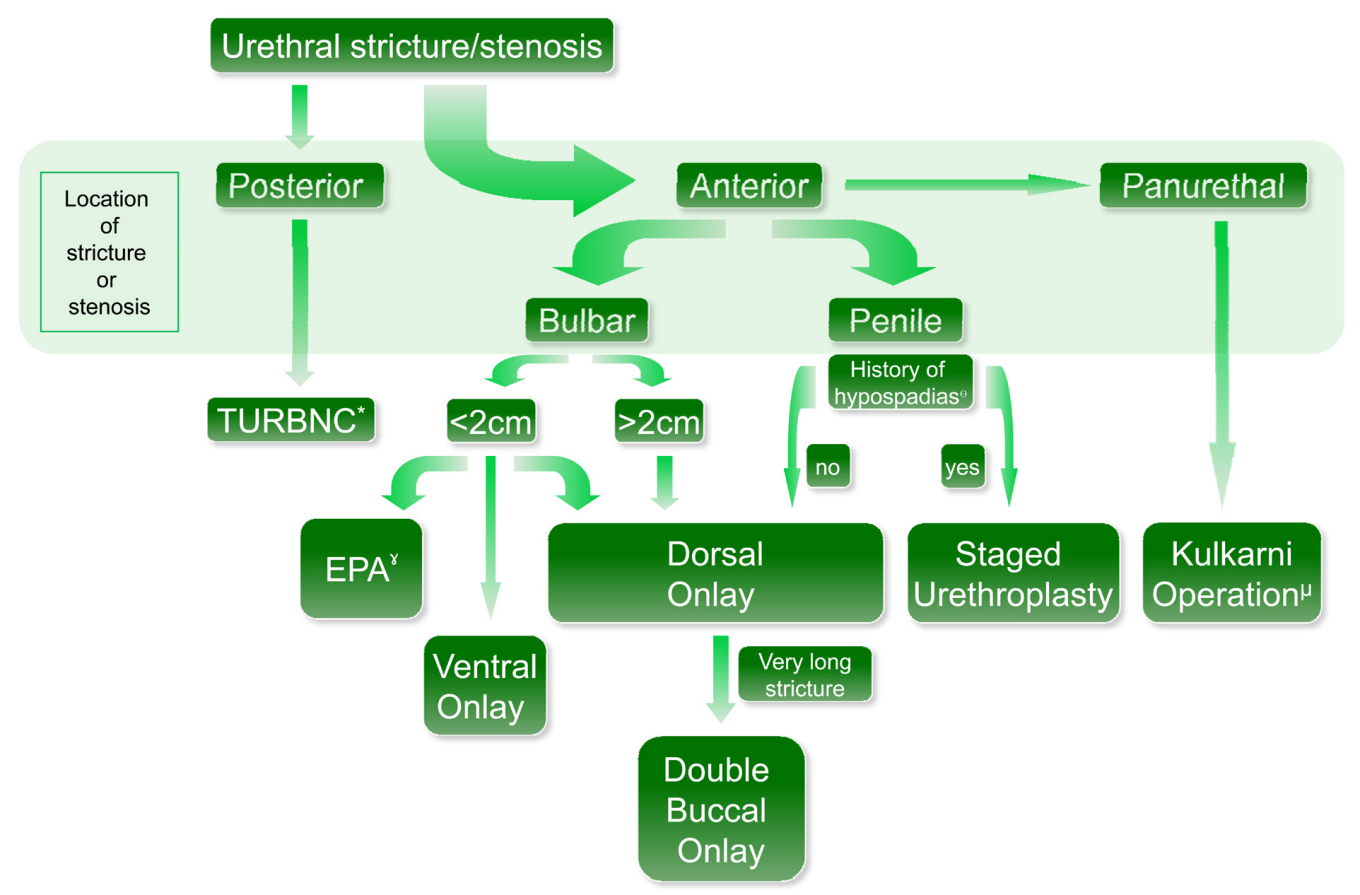
Urethral stricture treatment algorithm. * Consider mitomycin C instillation during the time of transurethral resection of a bladder neck contracture. ˠ Employ caution, as this technique has a high sexual complication rate. ɵ Also applicable in the event that urethra is completely obliterated. ^µ^ Dorsal onlay buccal urethroplasty with unilateral urethral dissection and penile inversion through a perineal incision.

This review will discuss the current management options for urethral stricture disease and the evolution of its treatment algorithm ( Figure 1). As we progress though different sizes and locations of urethral stricture, we will construct a treatment algorithm that focuses on which urethroplasty technique to employ and when to employ it. Additionally, there are new and exciting techniques in the realm of scar modulation and tissue engineering.

## Evaluation

Evaluation starts with a detailed history and physical examination. Men typically report obstructive symptomatology such as straining to void, weak urinary stream, and incomplete emptying. Patients occasionally experience bladder stones and frequent urinary tract infections. Uroflowmetry will show an obstructive/flattened voiding pattern with or without elevated post-void residual volumes. Cystoscopy may be useful to establish the diagnosis, as it is highly specific for urethral stricture ^4^. Retrograde or antegrade urethrography or both provide the length and location of the stricture and should be obtained prior to non-urgent intervention ^4, 5^. With this information, the urologist can make a well-informed decision regarding which method of treatment to use.

## Management

### Dilation and direct visualization internal urethrotomy

Dilation and direct visualization internal urethrotomy (DVIU) continue to predominate for urethral strictures. Among board-certified urologists, the most commonly performed procedures are dilation (93%) and DVIU (86%) ^6^. However, the failure rates of these minimally invasive strategies are well documented. One study found that the initial success rate of DVIU was only 9% after 1 to 3 years of follow-up ^7^. At 4 years, there is a nearly 0% chance of being stricture-free ^8^. Repetitive DVIU works no better. If a stricture has undergone three or more urethrotomies, the chance of lasting success approaches zero ^9^. If strictures are greater than 2 cm or multiple strictures exist, the results are also poor ^9^. Despite the limitations of dilation and DVIU, they may become more useful if new generations of currently experimental modalities for scar modulation prove successful (see the “Scar modulation, tissue engineering, and future directions” section).

### Urethroplasty

Urethroplasty is the definitive surgical treatment for urethral stricture and enjoys success rates of between 85 and 90% for simple procedures and about 80% for extremely complex repairs ^10^. Data show that when compared with urethral dilation or DVIU, open urethroplasty provides the best chance at definitive success. Urethroplasty is more cost-effective than repeated dilation or DVIU. Urethroplasty remains cost-effective even if it follows initial failed DVIU ^11, 12^ or is used as primary therapy ^13^. Both anastomotic urethroplasty and graft substitution urethroplasty have high long-term success rates, although the side effects may be different (see the “Excision and primary anastomosis urethroplasty” section).

Urethroplasty surgeries of one kind or another have been described for decades. Most of the advances in urethroplasty technique are in the optimization of the surgical treatment algorithm and in improvements in technique ( Figure 1). Correctly choosing which urethroplasty technique to use based on the characteristics of the stricture is key for a successful outcome.

Most strictures occur in the bulbar urethra (~50%), whereas 30% occur in the penile urethra. Finally, 20% of strictures are a combination of the two ^14^. As the bulbar urethra is the most commonly affected portion of the urethra, several methods of urethroplasty are used in this area. These primarily include excision and primary anastomosis (EPA) or substitution onlay grafting with buccal mucosa. Other, more complex flap and staged repairs are done but usually in failed or especially complex cases.

### Excision and primary anastomosis urethroplasty

Excision and primary anastomosis urethroplasty involves identifying the scarred urethral segment and excising it. After this, the healthy proximal and distal urethral ends are spatulated to ensure a patent anastomosis. This technique provides excellent long-term results of roughly 86% and a complication rate of 7% at 15 years ^15^. This technique is employed in the bulbar urethra. It has traditionally been used for strictures of 2 cm or less ^16^. Some experts suggest that it may be used for longer strictures with similar success rates, although greater reported numbers are needed to validate this statement ^17^. Some experts in the field of urethral reconstruction have suggested that EPA should be considered the optimal treatment for short bulbar strictures, citing long-term success rates of as high as 90 **–**95% ^18^. Others believe that the data support buccal urethroplasty for most strictures, citing lower penile complication rates ^19^.

A potential drawback to EPA is the risk of sexual complications (that is, ejaculatory dysfunction, cold or insensate glans, chordee, decreased penile length, and erectile dysfunction [ED]). ED is a well-documented potential outcome of anterior urethral surgery ^20^, which seems to be absent after buccal urethroplasty ^19^. Chordee (22%) and penile shortening (33 **–**44%) were found after EPA in one expert series ^17^. Barbagli *et al*. reported overall sexual complications after EPA of 22% ^21^. Specifically, these complications include decreased glans sensitivity, cold glans, soft glans during erection, and ejaculatory dysfunction ^21^. We found the rate of sexual complications after anastomotic urethroplasty to be higher than that for a matched cohort of buccal urethroplasty patients, although the buccal group had much longer strictures ^22^. It is notable that in a series specifically examining the sexual effects of buccal urethroplasty, these complications were absent ^19^. Owing to these side effects, some prominent urethral reconstructive surgeons have stopped performing this procedure, preferring buccal graft urethroplasty instead. However, it should be noted that EPA is not universally seen as more prone to sexual complications. A meta-analysis by Blaschko *et al*. found a 1% incidence of *de novo* ED ^23^. Recent studies have found that urethral transection may not be the cause of sexual dysfunction ^24^.

In an effort to avoid urethral transection and potential sexual side effects, non-transecting anastomotic urethroplasty techniques have been developed. Because the corpora spongiosum are not transected, the urethral blood supply is preserved. This potentially results in fewer sexual side effects ^25^.

### Graft onlay urethroplasty

Substitution graft urethroplasty is a common definitive treatment for urethral strictures. This technique can be used for short strictures as well as strictures of longer than 2 cm. The exposure for this method is similar to the exposure used in EPA. However, instead of excising the scarred urethral segment, a graft is placed beside the narrowed urethral lumen on one side and sutured to the contralateral side of the incised urethral lumen, effectively expanding the luminal diameter. Graft backing and blood supply are provided by the corpora cavernosum when the graft is placed dorsally, the corpus spongiosum when used ventrally, and the thin dorsal spongiosal tissue when placed as an inlay, as with the “Snodgraft” or “Asopa” techniques. Since the early 1990s, buccal mucosal graft has been the most used graft material for substitution urethroplasty, although lingual grafts can also be used to supplement or replace cheek mucosal grafts ^26– 28^. The overall success rates of onlay graft urethroplasty are about 90% when used in the bulbar urethra ^29^.

### Ventral onlay

For short bulbar strictures, a ventrally placed graft can be used. The spongiosal tissue is thick enough to allow easy closure of the tunica spongiosum over the graft and is robust enough to support the graft. This technique also allows for less dissection than dorsal grafting ^30, 31^. Some experts advocate dorsal grafting regardless of stricture position, citing less risk of diverticulum formation ^32^. They also believe that dorsally placed grafts may have a better graft support, although ventral grafts sit over highly vascularized spongiosal tissue ^32^. Several studies have shown similar success rates for ventral and dorsal grafting in the bulbar urethra ^33– 37^ ( Figure 1).

### Dorsal onlay

This method can be used for strictures in all locations of the urethra. It is especially useful in the thinner urethra of the distal bulb as it passes through the scrotum and in the very thin penile urethra. The dorsal approach to graft onlay urethroplasty is versatile and allows for the treatment of increasingly long strictures and even pan-urethral strictures. The urethra can be completely dissected from the corpora (Barbagli method) or mobilized unilaterally (Kulkarni method). When the urethra is mobilized unilaterally, the neurovascular supply of the urethra is also unilaterally preserved ^38^. A single buccal graft can be used to treat strictures of up to 4–7 cm in length, depending on oral dimensions. For longer strictures, we do not hesitate to use additional buccal grafts or lingual grafts ^38^.

### Combined dorsal and ventral buccal onlay: double buccal onlay

As strictures become longer (6 **–**8 cm range), the success rates of particularly ventral onlay urethroplasty fall. Simultaneous double buccal graft urethroplasty has been proposed to decrease failure rates in this case ^39^. A double graft repair may also be useful in near obliterated strictures, where sewing a ventral graft to a 2 **–**3 mm urethral plate may be both technically difficult and potentially associated with subsequent failure ^40^. This can be done as a dorsal inlay (Asopa) graft and a ventral onlay graft or, if the urethral plate is wide enough, as a combined true dorsal onlay and ventral onlay technique ^40^.

### Dorsal onlay buccal urethroplasty with unilateral urethral dissection and penile inversion through a perineal incision: Kulkarni technique

In 2000, a one-stage repair of very long and pan-urethral strictures was described by Kulkarni *et al*., who used buccal mucosal grafts and a penile inversion exposure ^41^. This repair has since been refined by using a unilateral mobilization to preserve the urethral blood supply ^38^. The penis is cleverly invaginated through a perineal incision, exposing the entire urethra up to the glans, similar to the exposure during urethrectomy ^41^. This optimized procedure is very satisfactory for the treatment of the worst sorts of strictures and supplants the former use of highly morbid, staged urethroplasty or fasciocutaneous flaps. This technique yields an 80 **–**83% long-term success rate despite being used in patients with features that might increase failure: very long strictures, lichen sclerosus, or failed previous urethroplasty ^42– 45^.

### Staged urethroplasty with or without buccal mucosa: Johanson technique

Owing to scarring and poor blood supply, patients who have previously undergone hypospadias repair, complete obliteration of the lumen, and dense lichen sclerosus-related strictures present particular challenges ^44, 46^. The two-stage technique with buccal graft can be used to treat these strictures with durable success. The first stage involves opening the urethra starting at the meatus through the strictured segment and securing the urethral edges to penile skin. The buccal graft is sutured to the lateral edges of the urethral plate, effectively widening the circumference. The second stage is closing or tubularizing the previously opened urethra ^45^. This technique is best used when previous hypospadias surgery was performed, when obliterated strictures are present, or severe lichen sclerosus is present ^45^. The utilization of buccal graft increases the success rate of this procedure from 33 to 85% and should be considered ^45, 47– 49^.

### Bladder neck contractures/post-prostatectomy anastomotic strictures

Bladder neck contractures most commonly occur after transurethral resection of prostate (TURP) and are distinct from post-prostatectomy anastomotic stenosis. The incidence is between 0.9 and 17% ^50– 52^. These contractures are often initially managed with dilation or urethrotomy and even with transurethral resection (transurethral resection of a bladder neck contracture). Vanni *et al*. have found that, when they are recurrent, radial urethrotomy and the injection of mitomycin C (MMC) yielded long-term success rates of 72% for one treatment and 89% after two treatments ^53^. Chen *et al*. report similar success rates for initial treatment when using bipolar incisions when compared with “cold” urethrotomy ^54^. Meanwhile, Ali and Ahmad *et al*. found that the use of MMC decreased the stricture recurrence and increased the time to recurrence in urethral strictures after DVIU ^55^. Interestingly, the TURNS Study Group found similar success rates (~75%) but also reported severe complications in 7% of patients ^56^. In that series, the dose of MMC was not standardized and often was much higher than doses suggested by Buckley *et al*. ^53^. We have observed that if MMC does not cure the stricture/contracture, it often prolongs the time to failure. Other studies suggest that deep lateral incisions alone provide good long-term results ^57^.

## Scar modulation, tissue engineering, and future directions

### Tissue engineering

Some of the most exciting areas of research in reconstructive urology are in tissue engineering and scar modulation. A more common form of tissue engineering is the use of acellular matrices. Essentially, these are protein scaffolds made mostly of collagen ^58^. These can be enhanced by adding autologous mucosal cells to the matrix, making a custom, biocompatible tissue-replacement matrix. The hope is that these matrices can be used to construct grafting material which can be used for urethroplasty. There have been several studies using these technologies as either ventral or dorsal grafts ^58, 59^. These techniques are exciting but are limited owing to inadequate native cellular ingrowth past 1 cm from the native urothelium. Engineered buccal mucosa is also being created. In this instance, buccal mucosa is taken from the patient and is grown in the laboratory. After a couple of weeks, sheets of tissue-engineered oral mucosa are created ^60^. Preliminary data show that tissue-engineered oral mucosa as grafting material has a success rate of 83% ^60^.

Another emerging technology which may have promise is the use of amniotic membrane to improve scarless healing after urethroplasty. Amniotic membrane is composed of basement membrane and stroma ^61^. It appears to possess active forms of over 250 cytokines and growth factors and has been useful in decreasing scarring and improving healing in a variety of tissues ^61– 63^. These biologically active cytokines may promote epithelial differentiation because of mitogenic factors, anti-inflammatory proteins, and anti-scarring effects ^61^. In a rabbit model, the use of amniotic membrane placed over buccal mucosa grafts provided significant epithelial transformation and may be a feasible adjunct at the time of buccal graft urethroplasty ^61^. An injectable form of Amniofix™ (MiMedx, Atlanta, GA, USA) has been used to decrease inflammation and promote healing. We are currently performing trials in patients with failed previous urethrotomy, who have an expected 0% chance of urethrotomy success.

### Scar modulation

One potential development that might revolutionize urethral stricture treatment is the advances of scar inhibitors that might be placed into the stricture after urethrotomy. There are several new technologies which are in the testing and pre-testing phases. Tacrolimus-impregnated coronary stents have been used because of the agent’s anti-proliferative and anti-inflammatory properties. It is hypothesized that such stents may be useful in urethral strictures ^64^. Similar coronary stents covered with paclitaxel have also been studied in a canine model demonstrating radiographic evidence of less luminal narrowing and histologic evidence of less tissue hyperplasia in an 8-week period ^65^. Systemic use of other chemotherapeutics such as docetaxel and rapamycin has also been investigated. In a rabbit model, the systemic docetaxel-receiving groups displayed less collagen deposition than did controls ^66^. In the rapamycin study, Chong *et al*. demonstrated that the rapamycin group had less luminal narrowing by retrograde urethrography and less fibroblastic activity histologically than did control groups ^67^. Although these trials are interesting, they may not have a future in humans, as the systemic use of both medications has undesirable effects.

Another potential scar-modulating technique could employ urinary catheters coated with anti-fibrosis agents. Krane *et al*. demonstrated that silicone catheters coated with halofuginone, a potent type-1 collagen inhibitor, prevented type-1 collagen deposition after experimental urethral injury in rabbits ^68^. Botulinum toxin A is a known scar modulator and has been shown to prevent facial scars ^69^. Khera, Boone, and Smith used botulinum toxin A after urethrotomy in just two patients with a short follow-up but had unexpected excellent results ^70^. This is an off-label use of Botox ^®^ (Allergan, Dublin, Ireland) and will be limited to stricture locations far enough away from the urinary sphincter that they will not cause incontinence.

## Conclusions

There have been great improvements in urethral surgical technique and optimization of the surgical treatment algorithm. Both EPA and graft onlay urethroplasty have high long-term success rates, but EPA has a controversial effect on sexual function and this is unacceptable to some prominent reconstructive urologists. Our treatment algorithm does include the option of EPA, but before employing this method, caution should be emphasized.

The experimental technologies mentioned above are exciting and ripe with potential. We are cautiously hopeful that these technologies will yield further improvements in the treatment of urethral stricture/stenosis.
